# Mechanism Based Quality Control (MBQC) of Herbal Products: A Case Study YIV-906 (PHY906)

**DOI:** 10.3389/fphar.2018.01324

**Published:** 2018-11-19

**Authors:** Wing Lam, Yongshen Ren, Fulan Guan, Zaoli Jiang, William Cheng, Chang-Hua Xu, Shwu-Huey Liu, Yung-Chi Cheng

**Affiliations:** ^1^Department of Pharmacology, Yale University School of Medicine, New Haven, CT, United States; ^2^College of Food Science and Technology, Shanghai Ocean University, Shanghai, China; ^3^Yiviva Inc., New York, NY, United States

**Keywords:** YIV-906, Chinese medicine, mechanism, quality control, chemical fingerprint, herbal products

## Abstract

YIV-906 (PHY906), a four-herb Chinese medicine formulation, is inspired by an 1800 year-old Chinese formulation called Huang Qin Tang which is traditionally used to treat gastrointestinal (GI) symptoms. In animal studies, it could enhance anti-tumor activity of different classes of anticancer agents and promote faster recovery of the damaged intestines following irinotecan or radiation treatment. Several clinical studies have shown that YIV-906 had the potential to increase the therapeutic index of cancer treatments (chemotherapy, radiation) by prolonging life and improving patient quality of life. Results of animal studies demonstrated five clinical batches of YIV-906 had very similar *in vivo* activities (protection of body weight loss induced by CPT11 and enhancement of anti-tumor activity of CPT11) while four batches of commercial–made Huang Qin Tang, HQT had no or lower *in vivo* activities. Two quality control platforms were used to correlate the biological activity between YIV906 and HQT. Chemical profiles (using analysis of 77 peaks intensities) obtained from LC-MS could not be used to differentiate YIV-906 from commercial Huang Qin Tang. A mechanism based quality control (MBQC) platform, comprising 18 luciferase reporter cell lines and two enzymatic assays based on the mechanism action of YIV-906, could be used to differentiate YIV-906 from commercial Huang Qin Tang. Results of MBQC could be matched to their *in vivo* activities on irinotecan. In conclusion, the quality control of an herbal product should be dependent on its pharmacological usage. For its specific usage appropriate biological assays based on its mechanism action should be developed for QC. Chemical fingerprints comparison approach has limitations unless irrelevant chemicals have been filtered out. Additionally, using a similarity index is only useful when relevant information is used. A MBQC platform should also be applied on other herbal products.

## Introduction

In human history Herbal Medicine is the oldest medicine. Herbs are the most important elements in different traditional medicines from different cultures around the world including Traditional Chinese Medicine, Ayurveda, Unani and Sidha. Many herbs are widely claimed to help a variety of disease or symptoms. In Asia and certain countries, herbal medicines are used as mainstream medicine for treating diseases. However, in many western countries, most herbal products are still being used as food supplements with low quality control standards. In order to promote herbal medicine as an accepted mainstream medicine in western countries for unmet needs, herbal products need to pass clinical trials with favorable and consistent clinical outcome. So far the FDA has only approved two botanical drugs: Veregen^®^ Ointment and Fulyzaq^®^ (crofelemer). FDA approved Veregen^®^ Ointment, a green tea extract as topical drug for genital warts in 2006. In 2012 the FDA approved Fulyzaq^®^ (crofelemer), an extract of the latex of the South American tree *Croton lechleri* for treating diarrhea in HIV patients in 2012 (Yeo et al., 2013). Quality control for Fulyzaq^®^ is relatively simple because it contains only purified oligomeric procyanidins and proanthocyanidins, which are polymers of (epi)catechin or (epi)gallocatechin.

Many herbal medicines are used as raw extracts with polychemicals because purification may lead to separate active compounds or lose their biological activity. In Traditional Chinese Medicine (TCM) formulations with multiple herbs are commonly used. Due to the chemical complexity of herbal products, it is extremely difficult to reproduce an herbal product with the same biological activity over time without knowing all active ingredients.

We are currently developing YIV-906 (formerly PHY906, KD018) as an adjuvant for cancer therapies. YIV906 is a standardized four-herb formula based on formula “Huang Qin Tang,” an 1800-year ago Chinese herbal formulation for numerous gastrointestinal (GI) symptoms, including diarrhea, nausea, and vomiting. These symptoms are common side effects of chemotherapy. YIV906 is composed of four herbs: *Glycyrrhiza uralensis* Fisch (**G**), *Paeonia lactiflora* Pall (**P**), *Scutellaria baicalensis* Georgi (**S**), and *Ziziphus jujuba* Mill (**Z**). YIV-906 was prepared using high-quality herbs selected by highly experienced herbalists and manufactured according to cGMP (current Good Manufacturing Practice) standards. Results from seven Phase (I/II to II) clinical trials with different batches of YIV-906 on 140 evaluable patients in Yale University and other institutions in United States suggested that there was no YIV-906 related toxicity with the used dosage; a clear indication of decreased G3/4 diarrhea, nausea, vomiting, and improved quality of life in those patients who received irinotecan, capecitabine or chemo-radiation were observed (Farrell and Kummar, 2003; Saif et al., 2010; Kummar et al., 2011; Saif et al., 2014). In preclinical studies YIV-906 reduced CPT-11-induced intestinal inflammation by inhibiting NFκB, COX-2, and iNOS while promoting intestinal stem/progenitor cell repopulation by stimulating the Wnt signaling pathway (Lam et al., 2010). YIV-906 also decreased GI toxicity from irradiation (Rockwell et al., 2013). YIV-906 could selectively alternate bacteria population of the intestines dependent on different treatment conditions (Lam et al., 2014). In tumors, YIV-906 was shown to enhance the anti-tumor activity of different classes of anti-cancer agents *in vivo* (Liu and Cheng, 2012). Detailed mechanism studies indicated that YIV-906 increased the anti-tumor activity of CPT-11 and Sorafenib by increasing apoptosis of tumor cells and promoting the polarization of macrophage to M1-like-type that assists tumor rejection in the tumor micro-environment (Wang et al., 2011; Lam et al., 2015). mRNA array results of colon 38 tumor following CPT-11+YIV-906 treatment suggested that YIV-906 could switch the immune status of tumor from chronic to acute inflammation associated with the up-regulation of IRF5, IFN, and JAK/STAT signaling (Wang et al., 2011). Overall, different batches of YIV-906 manufactured over a period of 15 years appeared to show similar biological activities in clinical and pre-clinical studies.

In this report, we compared different batches of YIV-906 with alternate commercial-made batches of Huang Qin Tang (HQT) for their biological activity on CPT11 of colon 38 tumor bearing BDF1 mice. We also compared correlation analysis based on chemical profiles, which is the most common quality control method used in botanical industry, against biological activities of “mechanism based quality control”(MBQC) could be used to differentiate clinical batches of YIV-906 from the commercial batches of HQT and matched to their biological activity *in vivo*.

## Materials and Methods

### *In vivo* Mouse Models

Murine Colon 38 cells (1-2 × 10^6^ cells in 0.1 ml phosphate-buffered saline, PBS) were transplanted subcutaneously into 4- to 6-week-old female BDF1 mice (Charles River Laboratories). After 10–14 days, mice with tumor sizes of 150–300 mm^3^ were selected. Unless otherwise indicated, treatment groups each consisted of five mice. Tumor size, body weight, and mortality of the mice were monitored daily. Tumor volume was estimated by using the formula length × width^2^ × π/6. Unless otherwise indicated, treatment groups each consisted of five mice. PHY906 (batches number 6, 7, 8, 10, 11 and F, 38, 39, 40 which are commercial Huang Qin Tang) were given orally (po) for 4 days [twice per day (b.i.d), 500 mg/kg at approximately 10:00 am and 3:00 pm], while CPT-11 (360 mg/kg) was administered intraperitoneal (ip) on Day 1. On Day 1, PHY906 was given 30 min prior to CPT-11 administration. In the control groups, mice were administered a vehicle, either PBS for i.p. administration or water for oral administration. Data was analyzed by two-way ANOVA (GraphPad Prism 6), The difference was considered to be statistically significant when ++ (*P* < 0.001), + (*P* < 0.05) and – (*P* > 0.05).

### LC-MS Analysis for Chemical Profiles of the Metabolites of PHY906

10 μl of 10 mg/ml herbal water extract of each sample was subjected to LC-MS analysis. Six times individual experiments were repeated for each sample. The LC-MS analysis was performed on an Agilent 1200 series HPLC coupled with AB SCIEX 4000 QTRAP mass spectrometer. The separation was conducted on an Agilent Zorbax C18HPLC Column (5 μm, 4.6 × 250 mm). The mobile phase is acetonitrile (A) and water with 0.1% formic acid (B) with linear gradient elution: 0 min, 5% A; 10 min, 20% A; 20 min, 25% A; 40 min, 30% A; 45 min, 35% A; 55 min, 45% A; 60 min, 70% A; 62 min, 90% A; 67 min, 90% A; 68 min, 5% A; and 75 min, 5% A. The flow rate is 1.0 mL/min, and the column temperature was set at 30°C, the detection wavelength was set at 230 nm. The mass spectrometer was operated in the negative modes and equipped with a electrospray ionization (ESI) source. Source parameters were as follow: sheath gas (nitrogen) flow rate and auxiliary gas (nitrogen) flow rate: 60 and 20 arbitrary units, respectively, capillary temperature: 400°C, heater temperature: 30°C, spray voltage was -3.8 kV. The instrument was operated from m/z 120–1000 Da in the full scan mode. Acquisitioning and processing of the data from the mass spectrometer was performed using Analyst 1.4.2^®^ Software, the peaks were compared and a clustering analysis was created by MZmine software.

### Mechanism Based Quality Control Platform

18 x Luciferase report cell lines for different signaling pathways were selected. Cells were seeded into half-area 96-well microplate at 20000 cells/well in 40 ul medium for overnight at 37°C 5% CO_2_ incubator. Different dosages of PHY906 water extracted from 750 μg/ml to 83 μg/ml were added to the cells and placed in a 37°C 5% CO_2_ incubator. After removing medium at 6 h, 10 μl of lysis buffer (Tris-HC 25 mM at pH7.8, DTT 2 mM, CDTA 2 mM, glycerol 10%, Triton X-100 1%) will be used to lyse the cells and 40 μl of luciferase reaction buffer (Tris-HCI 20 mM at pH7.8, NaHCO_3_ 1 mM, MgSO_4_ 2.5 mM, DTT 10 mM, Coenyzme-A lithium 60 μM, potassium luciferin 225 μM, ATP 250 μM) will be added for reading luminescence using a luminescence microplate reader. IC50 (concentration required to inhibit 50% of control) or EC50 (concentration required to achieve 50% of maximum activation) will be determined based on the dos-response curve. IC50 or E50 for each assay were determined from three independent experiments which were done in triplicate with 5 different doses. Methods for determining Cox-2 activity Assay and iNOS activity can be found in reference (Lam et al., 2010).

### Algorithm for Determining Correlation Coefficients

Graphpad Prism 6 software will be used to determine the correlation coefficients. Each raw input table represents different genes or different signal pathways. Each column represents different batches. Values of gene expression or IC50 or AC50 were input. “Column analyses” function of the software was selected for correlation analysis. Computing the correlation between each pairs of columns will be performed based on assuming a sample with Gaussian distribution. Pearson coefficients were calculated.

## Results

### All YIV-906 Batches but Not Commercial Batches of Huang Qin Tang Enhance Antitumor Activity of CPT11 While Reducing the Body Weight Loss Caused by CPT11

**FIGURE 1:**
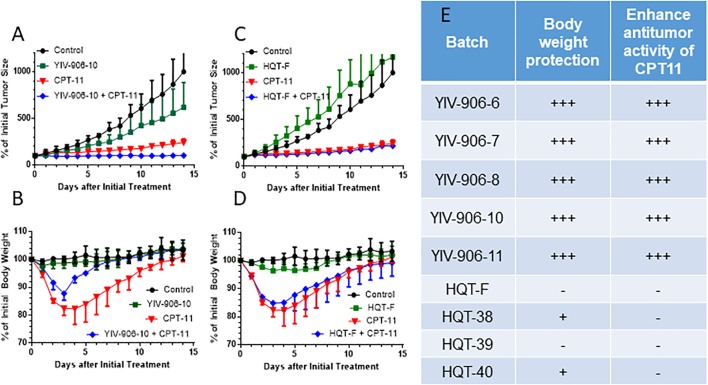
Comparison between *in vivo* activities among different batches of YIV-906 and HQT which is commercial Huang Qin Tang. **(A,B)**. Effect of YIV-906-10 **(A,B)** and HQT-F **(C,D)** on the anti-tumor activity of CPT11 and body weight protection of BDF1 bearing colon 38 tumor. **(E)** Summary of the effect of YIV-906 and HQT on the anti-tumor activity of CPT11 and body weight protection of BDF1 bearing colon 38 tumor. Details of experimental procedures are given in Section “Materials and Methods.”

We previously showed YIV-906-10 could enhance the action of CPT11 against colon-38 tumor growth while reducing body weight loss caused by CPT11. Here, we compared five different clinical batches (6, 7, 8, 10, and 11) of YIV906 which were manufactured separately over the span of 15 years with commercial batches of Huang Qin Tang, HQT (F, 38, 39, 40) on the biological activities of CPT11 on colon-38 tumor bearing BDF1 mice. Results indicated that YIV-906-10 and other batches YIV-906 enhanced the anti-tumor activity of CPT11 against colon-38 tumor growth (Figures 1A,E) while promoted body weight recovery following CPT11 treatment (Figures 1B,E). Commercial HQT (F, 38, 39, 40) had no or low *in vivo* activities for enhancing CPT11 action on colon-38 tumor growth (Figures 1C,E). Commercial HQT (F, 38, 39, 40) also had no activity in promoting body weight recovery following CPT11 treatment (Figures 1D,E). This result confirmed that YIV906 which manufactured apart 15 years could have very similar biological activities.

### Chemical Profile and Correlation Analysis for YIV-906 Batches and Commercial Batches of Huang Qin Tang Did Not Match Their Biological Activities on CPT11

**FIGURE 2:**
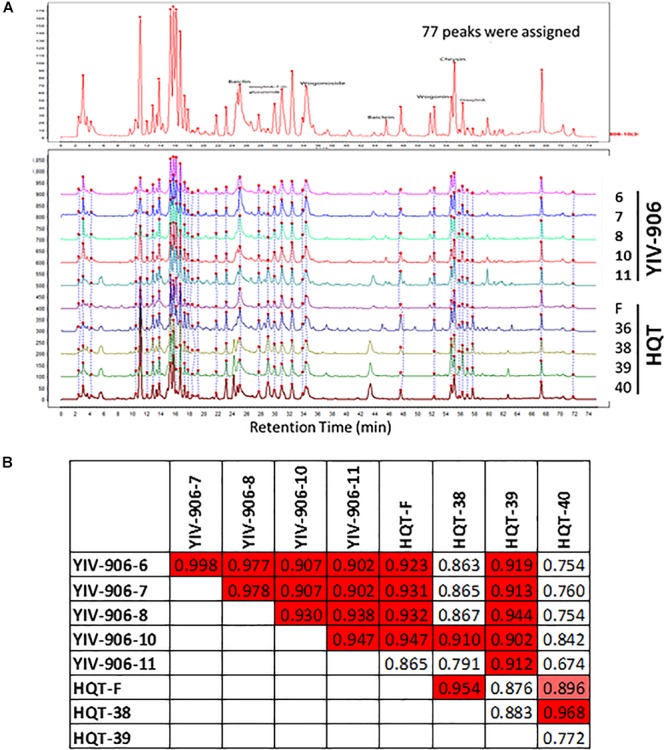
Chemical fingerprint analysis among different batches of YIV-906 and HQT which is commercial Huang Qin Tang. **(A)** Alignment of chemicals detected in LC-MS for YIV-906 and HQT which is commercial Huang Qin Tang. **(B)** Correlation analysis of chemical profiles of YIV-906 and HQT where 1 represents exactly the same and 0 is totally different. Details of experimental procedures are given in Section “Materials and Methods.”

Peaks from LC-MS profile were selected once their signals are significantly higher (5 folds higher) than the background (which was roughly about 5 × 10^4^). Totally 77 peaks, which were based on their specific ion pairs in the LC-MS spectra, could be selected from either YIV-906 or HQT. Peaks of the 77 peaks might or might be not commonly found in YIV-906 or HQT (Figure 2A). Totally integrated area of the 77 peaks was about 90% of total integrated area of all peaks of chemical profiles. Each corresponding peak of different batches of YIV-906 or HQT were de-noised and aligned using MZmine software (Figure 2A). When all 77 peaks were included for Pearson correlation analysis for each pair of samples, we did find that different pairs of YIV-906 batches demonstrated a strong positive similarity index (R from 0.9 to 0.99, red) (Figure 2B). However, based on their chemical profiles, HQT-F and HQT-39, which didn’t have any biological activity on CPT11 in animal, also showed a positive similarity to most batches of YIV-906. Therefore, using all detectable chemicals for quality control may lead to a false prediction.

### Mechanism Based Quality Control Platform and Correlation Analysis for YIV-906 Batches and Commercial Batches of Huang Qin Tang Matched Their Biological Activities on CPT11

Based on our previous animal and cell culture experiments, we know that YIV-906 had strong impact on inflammatory signaling via inhibiting NFkB, iNOS, COX2, IL6 and could promote tissue recovery by potentiating Wnt signaling. YIV-906 contains flavonoids which are known to have impact on hormone signaling and anti-oxidation property as well. Therefore, we selected 18 relevant luciferase reporter assays and two enzymatic assays, which were relevant to the mechanism action of YIV906, as our MBQC platform. We may not cover all biological activities for YIV-906 but all selected biological assays for YIV-906 could be used to explain its mechanism action for improving side effect caused by chemotherapy.

**FIGURE 3:**
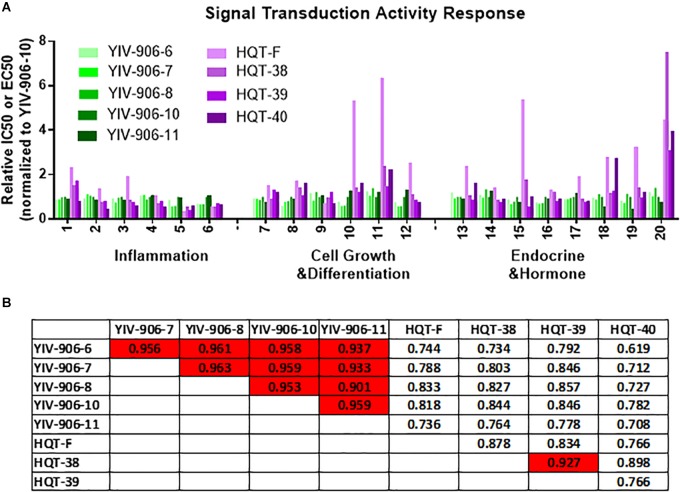
Signal transduction activity response analysis among different batches of YIV-906 and HQT which is commercial Huang Qin Tang. **(A)** Effect of different batches of YIV-906 and HQT on signal transduction activity response using different luciferase reporter cell lines and enzyme assays. **(B)** Correlation analysis of the signal transduction activity response for YIV-906 and HQT where 1 represents exactly the same and 0 is totally different. Details of experimental procedures are given in Section “Materials and Methods.”

We compared the signal transduction activity responses of clinical batches (6, 7, 8, 10, and 11) of YIV906 and commercial batches (F, 38, 39, 40) of HQT across these assays (Figure 3A). As compared to YIV-906-10, YIV-906 (6, 7, 8, and 11) showed very similar activities in different assays (Figure 3A). However, commercial HQT (F, 38, 39, 40) showed very low activities (with much larger EC50 or IC50) in certain assays when compared with YIV-906 (Figure 3A). Biological activities among the HQT batches were also very different (Figure 3A). When all these results from the assays of MBQC platform were analyzed using Pearson correlation for each pair samples, we found different batches (6, 7, 8, 10, and 11) of YIV-906 had quite high similarity (*R* > 0.9) (Figure 3B). As expected, HQT (F, 38, 39, 40) batches had lower similarity to YIV-906 (6, 7, 8, 10, and 11) batches (Figure 3B) (*R* < 0.9). Most importantly the correlation analysis based on the MBQC platform fitted results from animal experiments where all YIV-906 showed biological activity on CPT11 but not HQT. This method can be even further simplified using fewer bioassays.

## Discussion

In this study, we reported that different batches of YIV-906 manufactured over a period of 15 years had very similar biological activity on CPT11 in animals but not in commercial batches of Huang Qin Tang (HQT). We developed a MBQC platform to differentiate YIV-906 from HQT and predict their biological activities in animals. Chemical profile analysis based on all detectable chemicals of YIV-906 or HQT could not be used to differentiate them and may lead to false predictions for their biological activities.

Here, we showed that the clinical grade YIV-906 had better quality than commercial HQT because different batches of YIV-906 were manufactured according to cGMP protocol in which each manufacturing steps followed standard operation procedure. In addition, herbs for YIV-906 manufacturing were selected by very experience herbalists from defined source and specific season. Other HQT may use different sources of herbs and may not follow cGMP protocol. Some HQT also have different ratio of the four herbs from YIV-906. Therefore, different batches of YIV-906 had better consistency of their biological activities than other HQT.

In 2000, we proposed “phytomics” which covers both chemical and biological fingerprints to characterize herbal mixture (Tilton et al., 2010). However, the relevance of detected chemicals or biological responses to pharmacological activity of herbal mixtures could be an issue. This report highlights that chemical profiles analysis is not the ideal methodology for the quality control of herbal products.

For the past 20 years due to the available of many advanced analytical chemistry technologies, such as HPLC-MS, GC-MS, CE-MS, LC-NMR, NIR, NMR, 2D-IR in the market (Jiang et al., 2010; Song et al., 2013; Wang et al., 2013; Cheng et al., 2014), using chemical profile analysis as quality control became very popular in the botanical industry. People strongly believed that herbal products with similar detected chemical profiles should have similar biological activities. Even herbal pharmacopeia published by some countries heavily rely on one or two so called “key compound(s)” as the quality indication for many herbs. However, quality control dependent on using specific chemical detection can have many drawbacks. There is no single chemical detection method that can cover 100% of the chemicals in a given herbal product: Different chemical detection techniques have their advantages and limitations for detecting certain class chemicals. Many herbs or formulation of herbs have multiple active compounds, without knowing comprehensively all biological activity of all chemicals of these herbal products, including irrelevant chemicals, the analysis will mask the differences and interactions between different herbal preparations.

According to the Botanical Drug Development Guidance for Industry published by FDA in 2016, it is clear that the identification of each constituent of botanical product is impossible and identification of active constituents is not essential. Furthermore, the guidance suggests that biological assays should be developed for the active constituents which are not known or quantifiable before Phase 3 studies. Therefore, future quality control for herbal products should be focusing more on the biological activity of herb product based on their usage rather than their chemical profiles. With enough scientific knowledge on the mechanism action for different claims of a given herbal product, we can select relevant biological assays to establish a specific quality control platform for assessing the quality of the herbal product for the particular usage. The results from the quality control platform *in vitro* should be further validated using *in vivo* experiments.

In conclusion, the quality control of the herb should be depended on its usage. Appropriate biological assays should be developed for the QC of its particular usage. Unless irrelevant chemicals have been filtered out, chemical fingerprint analysis alone has notable shortcomings. Additionally, using a similarity index is only useful when relevant information is used and subject to bias. Unless all the active compounds of an herbal product have been identified, MBQC should always take precedent in the chemical profile analysis and collaboration between academia and industry could help further develop a rigorous MBQC platform. Our novel approach for quality control or CMC could be applied to other herbal medicines in order to ensure their biological activity.

## Ethics Statement

Animal experimental protocols were approved by Yale University Institutional Animal Care and Use Committee (IACUC). All animal experiments were carried out in accordance with an approved Yale University Institutional Animal Care and Use Committee (IACUC) protocol.

## Author Contributions

WL did luciferase reporter assays, correlation analysis, and wrote the manuscript. YR did LC-MS analysis. FG did luciferase reporter assays and enzymatic assays. ZJ did animal experiments. WC did correlation analysis and data processing for LC-MS. S-HL provided YIV-906 and wrote the manuscript. C-HX helped setting up LC-MS. Y-CC designed experiments and wrote the manuscript.

## Conflict of Interest Statement

Y-CC and S-HL are the co-inventors of YIV-906 for cancer treatment. Yale has the IP position. S-HL is employee of Yiviva which had licensed the IP of YIV-906 from Yale and Yale is a cofounder of the company. The remaining authors declare that the research was conducted in the absence of any commercial or financial relationships that could be construed as a potential conflict of interest.
